# Biological changes in auditory function following training in children with autism spectrum disorders

**DOI:** 10.1186/1744-9081-6-60

**Published:** 2010-10-16

**Authors:** Nicole M Russo, Jane Hornickel, Trent Nicol, Steven Zecker, Nina Kraus

**Affiliations:** 1 Auditory Neuroscience Lab, Department of Communication Sciences, Northwestern University, Evanston, IL, USA www.brainvolts.northwestern.edu; 2 Department of Pediatrics, Rush University Medical Center, Chicago, IL, USA; 3 Department of Behavioral Sciences, Rush University Medical Center, Chicago, IL, USA; 4 Department of Neurobiology and Physiology, Northwestern University, Evanston, IL, USA; 5 Department of Otolaryngology, Northwestern University, Evanston, IL, USA

## Abstract

**Background:**

Children with pervasive developmental disorders (PDD), such as children with autism spectrum disorders (ASD), often show auditory processing deficits related to their overarching language impairment. Auditory training programs such as Fast ForWord Language may potentially alleviate these deficits through training-induced improvements in auditory processing.

**Methods:**

To assess the impact of auditory training on auditory function in children with ASD, brainstem and cortical responses to speech sounds presented in quiet and noise were collected from five children with ASD who completed Fast ForWord training.

**Results:**

Relative to six control children with ASD who did not complete Fast ForWord, training-related changes were found in brainstem response timing (three children) and pitch-tracking (one child), and cortical response timing (all five children) after Fast ForWord use.

**Conclusions:**

These results provide an objective indication of the benefit of training on auditory function for some children with ASD.

## Background

Children with Pervasive Developmental Disorders (PDD), such as children with autism spectrum disorders (ASD), autism, Asperger's syndrome, or Pervasive Developmental Disorder - Not Otherwise Specified, demonstrate some level of impairment in the social and communicative use of language, social interactions, and imaginative and symbolic play, with an onset prior to the age of 3 years [1-3]. Some children with ASD have difficulties processing speech in background noise [4,5]. To this effect, emerging evidence suggests that the neural encoding of speech sounds may be impaired in these children [6-11]. Some children with ASD exhibit auditory brainstem processing deficits specific to speech stimuli [9,10], such as deficits in neural synchrony (timing) and phase locking (periodicity encoding; transcription of pitch contour), as well as degradation of the morphology of the responses in quiet and background noise, despite normal click-evoked brainstem responses. Reduced amplitude, delayed timing, and overall degraded morphology of cortical responses to speech syllables have also been reported in children with ASD relative to typically-developing (TD) children [6-8,11,12].

Several interventions targeting language, social skills, and auditory processing have been implemented for children with ASD (e.g., [13-18], see also the National Autism Center's National Standards Project report [19]). Given the variable nature of ASD, it is not surprising that treatment options and success vary across individuals. Thus, although studies show promise of success, further research is still needed to distinguish appropriate interventions for a specific child.

Fast ForWord Language (FFW; Scientific Learning Corp.; composed of Fast ForWord Language and Language to Reading) is a commercially available language training program consisting of seven games focusing on perceptual discrimination and language comprehension. The program provides auditory-focused training, including lessons in listening and sound sequencing, auditory attention, auditory discrimination, phoneme discrimination, and memory. Game sounds are spectrally and temporally altered to enhance cues important for speech discrimination and these enhancements are gradually reduced as a child progresses through the game. Games are completed when the child reaches an accuracy criterion (85%), which eventually leads to the advancement from the Language to the Language to Reading program. A retrospective study of 100 children with ASD who used FFW Language along with their regular intervention program showed that almost every child who completed training showed improvements in receptive and expressive language [20].While this study suggests that FFW training can benefit children with ASD with respect to language learning, objective neural indices of auditory function were not assessed and the impact of FFW on biological functions in children with ASD were not known.

This study evaluated the effectiveness of FFW for strengthening central auditory processing of speech sounds presented in quiet and background noise conditions in high-functioning children with ASD. Given the evidence from the retrospective study of children with ASD [20], in conjunction with the reports of improvements in central auditory processing after auditory training in both the brainstem [21-24] and cortex [21,22,25-28], and considering the auditory-based training components of FFW, we hypothesized that FFW training modifies the neural processing of sound in children with ASD. We predicted that children who completed FFW exercises would show improvement in the neural encoding of speech syllables, including faster response timing, greater fidelity of the response relative to the stimulus, and more accurate pitch encoding over time. Here we present case studies of the biological impact of FFW in five children with ASD.

## Methods

The Institutional Review Board of Northwestern University approved all research; informed consent by the parent or guardian and child assent were obtained. The Institutional Review Board ensures that all research is conducted in compliance with the Helsinki Declaration. Children and their families were invited to the research laboratory prior to the onset of the study to become acclimated with the testing location and equipment prior to experimental data collection. Data were compared to test-retest data from six children with ASD who did not participate in an active intervention; informed consent and assent was also obtained from these participants.

### Participants

Families who participated in previous, more comprehensive research studies of neural processing of speech and prosody in children with ASD [9-11,29] were invited to participate in this study. All children were required to have (a) a formal ASD diagnosis made by a child neurologist or psychologist and to be actively monitored by their physicians and school professionals at regular intervals; (b) the absence of a confounding neurological diagnosis (e.g., active seizure disorder, cerebral palsy); (c) normal peripheral hearing as measured by air threshold pure-tone audiogram (≤20 dB HL for octaves between 250 and 8000 Hz) and normal click-evoked auditory brainstem responses (wave V latency, at 80.3 dB SPL [16,19,20]); (d) a full-scale mental ability score ≥85 as assessed by the Wechsler Abbreviated Scale of Intelligence [30]; and (e) a Core Language score ≥80 as assessed by the Clinical Evaluation of Language Fundamentals [31]. A control group of six boys with ASD (mean age = 9.00 years, SD = 1.549) who completed the entire test protocol, but who did not express interest in participating in the intensive intervention, agreed to be retested on the original protocol. The control data were used to establish test-retest reliability of the test measures. Cortical response data for control participant C3 were unavailable so the resulting mean values for cortical response measures are calculated from the remaining five subjects. See Table 1 for subject information.

**Table 1:** Participant characteristics.

Subject	FFW Training Duration (days)		Total Training Duration	Pre-post Test Duration (months)	Other Interventions
	Language	Language to Reading			
S1	33	23	56	16	Speech and dietary therapy, previously occupational therapy
S2	18	19	37	8	Previously occupational, speech, and music therapy
S3	12	48	60	6	Social skills group, therapeutic day school, previously biofeedback and occupational therapy
S4	12	35	47	9	Speech, occupational, music, and dietary therapy, language group and therapeutic exercise
S5	25	36	61	7	Speech therapy, previously occupational therapy and social skills training
C1				19	Speech and occupational therapy
C2				15	Speech therapy and special education programming, formerly occupational therapy
C3				14	Speech and occupational therapy
C4				14	Occupational therapy, social skills group
C5				12	Speech and occupational therapy
C6				10	Occupational and active music therapy

Training duration, pre-test to post-test interval, and additional interventions are listed for each participant.

Five boys (mean age = 9.40 years, SD = 1.517) underwent auditory training through FFW for a period of 5-10 weeks (mean = 7.45 weeks). Duration was determined by each child's own progress through the Language and Language to Reading components of the program. Participants and their families each began by receiving instruction from a trained supervisor of the Scientific Learning Corporation and then transitioned to training at home once it was determined they understood the program. Training was completed when the child reached an 85% completion rate (percentage = number of levels through which the child advanced) and thus duration varied across cases. Each child in the trained group began training within one year of their first session (average = 5.1 mos, SD = 4.29) and were tested again approximately 1 month after having completed training (average = 34.6 days, SD = 18.11). Participants were self-selected and, as a consequence, the presence or degree of deficit in auditory neurophysiological profiles before training was not controlled. See Table 1 for subject information.

The control and trained groups were not significantly different in age (Mann-Whitney U = 12.5, p = 0.644), IQ (Control mean: 104; Trained mean: 111.8; U = 11, p = 0.464), or language ability (Control mean: 97.33; Trained mean: 102.8; U = 12.5, p = 0.644). Further, groups did not differ significantly on test-retest duration (Control mean: 14 months; Trained mean: 9.2 months; U = 5, p = 0.067), although the trained group tended to have a shorter test-retest interval than the control group.

### Neurophysiological testing and data processing

The auditory brainstem response was collected to both a 40 ms synthetic "da" stimulus which was produced in KLATT [32] and two 230 ms "ya" syllables with linearly ramped fundamental frequency (F_0_) contours (130 - 220 Hz ascending; 220 - 130 Hz descending) that were digitally manipulated in Praat [33]. The "da" stimulus was presented in quiet (80 dB SPL) for brainstem recordings and in quiet and white background noise (+5 dB SNR) conditions for the cortical response recording. The "ya" stimuli were presented in quiet only (60 dB SPL), and were used only to collect brainstem responses. All stimuli were presented via ear inserts (brainstem "da": ER-3A, Natus Medical Inc.; brainstem "ya" and cortical "da": ER-3, Etymotic Research, Elk Grove Village, IL, USA) and responses were collected with vertical electrode montages (forehead ground, active Cz, ipsilateral earlobe reference). Brainstem responses to "da" were collected using the Bio-Logic Navigator Pro system at a 6856 Hz sampling rate. Responses were bandpass filtered from 100-2000 Hz (12 dB/octave slope), and trials with amplitude greater than ±23.8 μV were rejected. Three thousand accepted sweeps from each polarity were collected and averaged to form the final averaged response of 6000 sweeps. Brainstem responses to "ya" were digitized at 20,000 Hz by NeuroScan Acquire and bandpass filtered from 80-1000 Hz (12 dB/octave slope). Artifact rejection was ±35 μV. Two replications of 2400 sweeps (1200 each polarity) were collected for each stimulus. Cortical responses to "da" were collected through NeuroScan Acquire at a 2000 Hz sampling rate, bandpass filtered from 0.5-100 Hz (12 dB/octave slope), and artifact rejected at ±65 μV. Approximately 1000 accepted sweeps were collected. An additional electrode on the superior canthus of the left eye was used to monitor eye blinks. Additional details regarding stimuli, brainstem and cortical response collection and analysis can be found in previous reports using identical stimulus and recording parameters [9-11,34].

The measures employed in the current paper were limited to subcortical onset response timing and cortical response timing in quiet and noise. Response timing is represented through peak latencies; for the onset response to "da" Waves V and A and for the cortical response to "da" in quiet and noise P1' and N1'. Additionally, subcortical pitch tracking in response to "ya" (Frequency Error for F_0 _and a composite measure comprising Frequency Errors for F_0 _and the second harmonic (H_2_) and Pitch Strength) was assessed. These measures have been shown to be significantly impaired in children with ASD relative to typically-developing children and represent the most robust differences between the groups [9-11]. All data analyses were automated using routines coded in Matlab 7.4 (The MathWorks, Inc., Natick, MA).

### Statistical analyses

Responses from the trained group were judged against the amount of change expected due to chance by comparing each individual's data with the average amount of change seen in the control group. In order for a change to be deemed significant, it was required to be greater than 1 standard deviation (SD) from the mean control change for that measure (see Table 2). This criterion is often employed for clinical diagnoses. The probability of showing improvement on any one measure by chance is ~16%; the probability of showing concurrent improvement on two measures is ~2.6% and three measures is ~0.4%. Therefore, improvement on two or more measures would occur in less than ~5% of the population simply due to chance, a standard alpha level for statistical analyses. Change was calculated as post-test value minus pre-test value and, for all variables but the composite pitch tracking measure, smaller numbers indicated more typical function (i.e., shorter latencies, less frequency error), so negative values indicated an enhancement in encoding. Because the pitch tracking composite was an average of values z transformed against the typically-developing (TD) mean (see [10]), enhanced encoding would be indicated by an increase in score. Additionally, subjects' responses were compared to previously-established norms in TD children to determine normalcy [9-11]. A criterion of 1.6 SD was used, which includes ~95% of individuals in a population.

**Table 2:** Group data from the untrained control group.

Measure	Pre-test Mean (SD)	Post-test	Change
Wave V Latency (ms)	6.59 (0.24)	6.67 (0.21)	0.07 (0.08)
Wave A Latency (ms)	7.54 (0.35)	7.69 (0.25)	0.12 (0.15)
Frequency Error (Hz)	7.69 (2.31)	8.51 (2.54)	0.65 (3.35)
Pitch Tracking Composite (z score)	-0.194 (0.62)	-0.291 (0.95)	-0.10 (1.09)
P1' Latency in Quiet (ms)	167.8 (34.59)	166.6 (24.72)	-1.20 (16.48)
N1' Latency in Quiet (ms)	255.8 (43.16)	258 (34.24)	2.20 (25.82)
P1' Latency in Noise (ms)	169.4 (20.36)	166.7 (22.88)	-2.70 (13.70)
N1' Latency in Noise (ms)	237.3 (45.83)	268.8 (24.00)	31.50 (49.84)

Means and standard deviations are reported for each measure at pre-test and post-test as well as change (post-pre) for control children. Cortical response measures were available for only five of the control children.

The FFW program does not issue skill level or percent correct scores at the onset or upon completion of the program, precluding quantitative analyses of the behavioral changes. Because the program is adaptive, success is determined by "completion" of the training exercises. Individual progress and associated changes are discussed below for each participant.

## Results

### Subject 1

**Figure 1:**
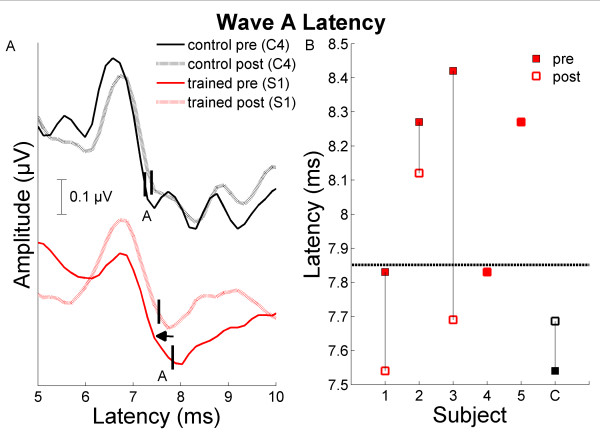
**Brainstem timing change in response to "da"**. A) Wave A of the response is plotted for trained subject S1 (red) and one control child, C4 (black), for both pre- (solid) and post- (dashed) test. The peaks chosen for Wave A are marked. B) Drop line plot of wave A latencies for all trained children (red) and the mean of control children (C, black) for both pre- (solid) and post- (open) test. The criterion for normalcy (1.6 SD later than the typically-developing mean) is plotted as a dashed line.

**Figure 2:**
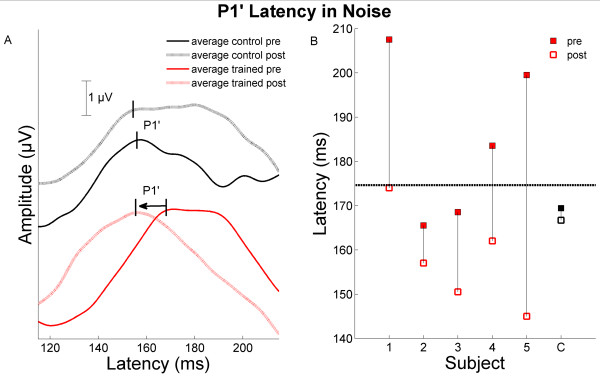
**Cortical timing change in response to "da" in background noise**. A) P1' of the response to "da" in background noise is plotted for the average of the trained children (red) and the average of the control children (black) for both pre- (solid) and post- (dashed) test. The peaks chosen for P1' are marked. B) Drop line plot of P1' latencies in noise for all trained children (red) and the mean of control children (C, black) for both pre- (solid) and post- (open) test. The criterion for normalcy (1.6 SD later than the typically-developing mean) is plotted as a dashed line.

S1 was clinically diagnosed with autism. Relative to untrained children with ASD, S1 showed improvements on subcortical and cortical response timing, but not pitch tracking. Although S1 did not demonstrate impaired brainstem processing relative to TD children at pre-training, response timing of Wave A became faster after training (improving from 1.5 SD above the TD mean to 0.25 SD above the TD mean (Figure 1B; [9]). Additionally, the cortical P1' response in background noise occurred earlier after training, improving from 3.9 SD to 1.6 SD (Figure 2B; [11]). On FFW Language, S1 showed consistent progress on temporal auditory processing tasks, but demonstrated persistent working memory difficulties continuing into Language to Reading.

### Subject 2

S2 was clinically diagnosed with Asperger's syndrome. Like S1, S2 improved on subcortical and cortical response timing, and also approached a significant change in the pitch tracking composite relative to the control group. S2 did show marked delay in wave A timing prior to training (3.4 SD above the TD mean), but moved to within 2 SD after training (See Figure 1B). In the cortical responses, P1' and N1' latencies in quiet became earlier (improving by 1.3 SD and 1.7 SD, respectively). He demonstrated consistent progress through the Language and Language to Reading exercises and completed training at a rapid pace (see Table 1). Behavioral speech sound discrimination and temporal processing were both noted as mild deficits at the onset of training and showed minor improvements according to the FFW report.

### Subject 3

S3 was clinically diagnosed as having an ASD and, relative to the control group, improved on subcortical and cortical response timing, with no improvement in pitch tracking. Although S3 demonstrated abnormal wave A timing (4.7 SD outside the TD mean) at pre-test, his response fell within normal limits after training (0.91 SD, see Figure 1B). S3 also demonstrated poor cortical processing prior to training, with faster P1' timing in quiet and background noise after training (changes of 2.2 SD and 1.3 SD, respectively; see Figure 2B). S3 progressed rapidly through FFW Language and proceeded consistently through Language to Reading.

### Subject 4

**Figure 3:**
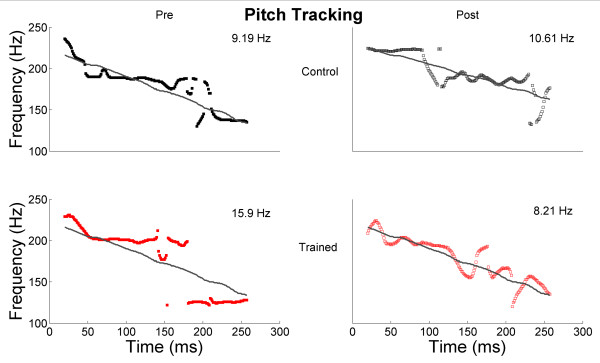
**Pitch-tracking change in response to "ya"**. Pitch-tracking curves are plotted for control subject C6 (black) and for trained subject S4 (red) for pre- (left, solid) and post- (right, dashed) test. Thin gray lines are the stimulus' F_0 _trajectory; thick lines are the response's F_0 _trajectory. Average frequency error values are plotted.

S4 was clinically diagnosed with Asperger's syndrome. Relative to the control group, S4 improved on cortical response timing, as well as subcortical pitch tracking accuracy. This participant showed marked impairment in both brainstem processing of "da" and pitch contour of "ya" and significant improvement in pitch processing after training relative to the control group (see Figure 3). S4 was a member of a previously reported pitch-tracking 'out' group (see [10]) as calculated by a composite score of Frequency Error of the fundamental frequency, Pitch Strength, and Frequency Error of the second harmonic in response to the "ya" stimulus. After training, S4 fell within normal limits on this measure, improving from 1.9 SD to 0.95 SD from the TD mean, although his improvement was just shy of significant relative to the control children. He did significantly improve on the Frequency Error of F_0 _when compared to the control group change. The improvements in pitch tracking seen in S4 are of particular interest because the majority of FFW modules do not specifically target frequency discrimination or pitch identification. While it is possible that the pitch-tracking deficits previously found in children with ASD [10] may be ameliorated or improved without specifically targeting frequency discrimination or pitch processing, according to parental report S4 was also participating in active music training throughout the study, which may have influenced pitch tracking improvements. Among cortical response measures, only P1' latency in background noise became earlier after training (a change of 1.5 SD; see Figure 2B). This subject progressed consistently on all FFW exercises, including Language and Language to Reading. A cautious relationship may be made between his consistent behavioral progression and neurophysiological improvements.

### Subject 5

S5 was clinically diagnosed as having an ASD, without a more specific distinction. S5 showed improvements in cortical response timing relative to the control group, with no change in subcortical measures of timing or pitch tracking. He demonstrated poor transcription of "da," poor pitch tracking, and poor cortical encoding prior to training. Although brainstem measures did not change, he did show faster cortical timing after training; P1' latencies in quiet and background noise decreased significantly (changes of 1.8 SD and 3.8 SD, respectively; see Figure 2B). Unfortunately S5 did not complete either FFW Language or Language to Reading and struggled with frustration throughout training. He was noted to have temporal auditory processing, speech sound discrimination and working memory problems, consistent with his pre-training brainstem and cortical deficits. Despite his improvement on cortical response latencies, his lack of improvement on brainstem measures may have been due to his inability to complete the full training protocol.

## Discussion

Although the present sample size is small, training appeared to have beneficial effects on some aspect of the neural transcription of speech in all children. Three children improved on the subcortical transcription of "da" in quiet and one child improved on pitch tracking relative to the change seen in the control children. Of the four trained children who showed brainstem improvements, two had poor transcription of "da" and/or "ya" at the pre-test relative to TD children and approached or reached normal limits after training. Further, each of the five children who underwent FFW training improved on at least one measure of cortical speech processing relative to the control group, with response timing improving in both quiet and noise for some children. This study is the first to report malleability of the *onset *response to "da" with short-term training, plasticity of subcortical pitch tracking of the frequency contour in the "ya" stimuli, and cortical response changes in children with ASD.

Because of our limited sample size and the variability in degree of autism severity and spectrum diagnosis within our group, we are unable draw specific conclusions with respect to children with autism versus Asperger's syndrome. It is important to note that all of the children in the study were high functioning with normal intelligence and language abilities. Thus, the differentiation of diagnosis and language ability had less impact on the findings of this study, as the focus was centered on identifying neural improvements in auditory processing after training with FFW, rather than expected behavioral improvements (which were not empirically evaluated in this study). That a pattern of improvement was identified even in this heterogeneous spectrum group suggests a broader application of the training to children on the spectrum.

## Conclusions

This study shows initial evidence of the efficacy of directed auditory training for improving auditory processing in a population of children with ASD. The biological changes identified in the current study were accompanied by consistent and successful progression through FFW training. Although a correlation between days of training and neural improvements was not identified, extended use of FFW or longitudinal testing may reveal more pronounced changes in brainstem function and also behavior improvements after a delay period. This proposition is consistent with previously published reports indicating that physiological changes can precede behavioral learning [35,36]. Our findings are also consistent with previously reported auditory training-associated physiological changes in clinical populations [21,23,28,37]. These findings suggest that computer-based auditory training programs may benefit some children with ASD by impacting biological processes.

Continuation of this work with a larger cohort, more specific inclusion criteria for diagnosis, and an active-control group would be important to replicate and further validate these preliminary findings. Future investigations may also evaluate which brainstem deficits are *predictive *of gains in response to auditory training programs, and what types of *behavioral *improvements may be associated with training both immediately following training and after a delay period. Thus, a study that establishes the long-term maintenance of these training effects and their consequences on language function would provide valuable information for both families of children with ASD and practitioners who treat these children.

## Competing interests

The authors declare that they have no competing interests.

## Authors' contributions

NR developed the study design, collected and processed the data, conducted and interpreted the statistical analyses, and prepared the manuscript. JH collected and processed the data, conducted and interpreted the statistical analyses, and prepared the manuscript. TN assisted with the data processing, statistical analyses, and interpretation and reviewed the drafts of the manuscript. SZ provided consultation with respect to statistical methods and interpretation and also reviewed the drafts of the manuscript. NK oversaw all aspects of the study, provided additional statistical consultation and also reviewed the drafts of the manuscript. All authors read and approved the final manuscript.
